# Sequence diversity of dengue virus type 2 in brain and thymus of infected interferon receptor ko mice: implications for dengue virulence

**DOI:** 10.1186/s12985-016-0658-4

**Published:** 2016-11-30

**Authors:** Priya Dhole, Emi E. Nakayama, Akatsuki Saito, Kriengsak Limkittikul, Supranee Phanthanawiboon, Tatsuo Shioda, Takeshi Kurosu

**Affiliations:** 1 Research Institute for Microbial Diseases, Osaka University, Osaka, Japan; 2 Faculty of Tropical Medicine, Mahidol University, Bangkok, Thailand; 3 Center for Emerging Infectious Diseases, Kohn Kaen University, Kohn Kaen, Thailand; 4 Department of Virology I, National Institute for Infectious Diseases, Tokyo, Japan

**Keywords:** Dengue virus, Mutation, Genome, Virulence specificity, Mouse model

## Abstract

**Background:**

We previously reported that a clinical isolate of dengue virus (DENV) is capable of causing acute-phase systemic infection in mice harboring knockouts of the genes encoding type-I and -II interferon IFN receptors (IFN-α/β/γR KO mice); in contrast, other virulent DENV isolates exhibited slow disease progression in this mice, yielding lethal infection around 20 days post-infection (p.i.). In the present study, we sought to clarify the dynamics of slow disease progression by examining disease progression of a type-2 DENV clinical isolate (DV2P04/08) in mice.

**Methods:**

The tissue distributions of DV2P04/08 in several organs of infeted mice were examined at different time points. Whole genome viral sequences from organs were determined.

**Results:**

At day 6 p.i., high levels of viral RNA (vRNA) were detected in non-neuronal organs (including peritoneal exudate cells (PECs), spleen, kidney, liver, lung, and bone marrow) but not in brain. By day 14 p.i, vRNA levels subsequently decreased in most organs, with the exception of thymus and brain. Sequence analysis of the whole genome of the original P04/08 and those of viruses recovered from mouse brain and thymus demonstrated the presence of both synonymous and non-synonymous mutations. Individual mice showed different virus populations in the brain. The vRNA sequence derived from brain of one mouse was nearly identical to the original DV2P04/08 inoculum, suggesting that there was no need for adaptation of DV2P04/08 for growth in the brain. However, quasispecies (that is, mixed populations, detected as apparent nucleotide mixtures during sequencing) were observed in the thymus of another mouse, and interestingly only mutant population invaded the brain at a late stage of infection.

**Conclusions:**

These results suggested that the mouse nearly succeeded in eliminating virus from non-neuronal organs but failed to do so from brain. Although the cause of death by DV2P04/08 infection is likely to be the result of virus invasion to brain, its processes to the death are different in individual mice. This study will provide a new insight into disease progression of DENV in mice.

**Electronic supplementary material:**

The online version of this article (doi:10.1186/s12985-016-0658-4) contains supplementary material, which is available to authorized users.

## Background

Recent climate change and urbanization have increased the risk of vector-borne disease [1–3]. Viruses of the genus flavivirus, which consist of positive-stranded RNA viruses, are transmitted by arthropod vectors and are responsible for many emerging and re-emerging infectious diseases [4]. Flaviviruses includes dengue virus (DENV), Japanese encephalitis virus, West Nile virus, tick-born encephaitis virus, and Zika virus [5, 6]. These viruses lead to diverse manifestations, ranging from mild fever and arthralgia to severe hemorrhage and encephalitis [7]. DENV infection is a major public health problem in tropical and subtropical areas of the world, resulting in annual totals of approximately 390 million DENV infections and approximately 500,000 deaths [4, 8]. DENV is transmitted by *Aedes aegypti* and *Aedes albopictus* [5]. Clinical manifestations of DENV infections range from fever in classical dengue fever (DF) to dengue haemorrhagic fever (DHF), which is characterized by plasma leakage and thrombocytopenia. Severe cases of DHF can lead to hypovolemic shock, so-called shock syndrome (DSS) [9].

DENVs possess a single-stranded RNA genome of approximately 10.7 kb [10]. The genome consists of a single long open reading frame, flanked by 5`- and 3`- untranslated regions (UTRs), that encodes a single polyprotein. This polyprotein is cleaved co- and post-translationally to yield mature structural and non-structural proteins [11]. The structural proteins include the envelope (E), membrane (M), and capsid (C) proteins, and the non-structural proteins include NS1, NS2A, NS2B, NS3, NS4A, NS4B, and NS5 [12, 13]. DENV exists as quasispecies, a complex mixture of genetically distinct but closely related variants, reflecting the error-prone nature of the DENV RNA-dependent viral RNA polymerase [14]. This property often provides an advantage to the virus by producing escape mutants capable of evading the immune system or drug therapy [14], and such variants also often play an important role in disease progression [14, 15]. DENV derived from patients have been shown to encompass populations with large sequence diversity [16].

Because the mechanism of severe disease remains obscure [17–19], development of an appropriate animal model reflecting DENV clinical manifestations is essential. While wild-type strains of mice do not show DHF-like symptoms upon DENV infection, recent work showed that, AG129 mice, which lack (IFN)-α/β and –γ receptors, can serve as a host for a model of severe dengue [20–23]. However, most DENV isolates do not induce DHF-like symptoms even in these knock out (KO) mice; only a subset of DENV isolates provide lethal infection in this background [24]. In the present study, we studied the infection dynamics of the DV2P04/08 clinical isolate, which causes lethal infection but exhibits slow disease progression in IFN-α/β/γR KO mice. Full genome sequence analysis demonstrated distinct in vivo evolution of DENV in different organs following infection of this mouse host strain.

## Methods

### Mice

IFN-α/β receptor KO mice and FcγRIIB receptor KO mice were the kind gifts of (respectively) Prof. Ken J, Ishii, Laboratory of Vaccine Science, WPI Immunology Frontier Research Centre (IFReC)/Laboratory of Adjuvant Innovation, National Institutes of Biomedical Innovation, Health and Nutrition and Prof. Toshiyuki Takai, Department of Experimental Immunology, Institute of Development, Aging and Cancer, Tohoku University. IFN-γ receptor KO mice were purchased from Jackson Laboratory. IFN-α/β/γR KO mice were obtained by breeding IFN-α/β receptor KO mice × IFN-γ receptor KO mice. IFN-α/β/γR/FcγRIIB KO mice were further obtained by breeding IFN-α/β/γR KO × FcγRIIB receptor KO mice. All IFN-receptor and Fcγ receptor KO mice were bred and maintained under specific-pathogen-free conditions in the animal facilities of the Research Institute for Microbial Diseases (RIMD), Osaka University (Osaka), Japan. After breeding, 4- to 5-week-old male and female mice were used for viral quantification and survival experiments, respectively.

### Virus and mouse infection

DV2P04/08 was a clinical isolate gifted by Dr. Kriengsak Limkittikul, Faculty of Tropical Medicine, Mahidol University, Thailand. Initially, a few generations of the virus were obtained by passaging in the C6/36 mosquito cell line. However, in order to augment the titer, the subsequent 2–3 generations were obtained by passaging in Vero cell lines. The titer of DV2P04/08 was determined by the focus forming assay [25]. We employed different titers of DV2P04/08 (3.5 x 10^6^ ffu, 3.5 x 10^5^ ffu, and 3.5 x 10^4^ ffu) for the IFN-α/β/γR/FcγRIIB and IFN-α/β/γR KO mouse survival experiments, while a titer of 2.4x10^6^ ffu was used for in vivo quantification of the virus production after infection of IFN-α/β/γR/FcγRIIB KO mice. Trained laboratory personnel performed anesthesia of mice via intraperitoneal injection of a mixture of medetomidine, midazolam, and butorphanol prior to injection of virus and (at a separate time point) euthanasia of mice by cervical dislocation. For the survival experiments, IFN-α/β/γR/FcγRIIB KO mice were injected intraperitoneally with 1 ml of a DV2P04/08 viral suspension at 3.5 x 10^5^ ffu/ml or 3.5 x 10^4^ ffu/ml. IFN-α/β/γR KO mice were injected intraperitoneally with 1 ml of a DV2P04/08 viral suspension at 3.5 x 10^6^ ffu/ml or 3.5 x 10^4^ ffu/ml. Following infection, both the IFN-α/β/γR/FcγRIIB KO and IFN-α/β/γR KO mice were observed daily for any conspicuous clinical manifestations. In a separate experiment, in vivo systemic viral titration was performed by injecting IFN-α/β/γR/FcγRIIB KO mice intraperitoneally with 1 ml of a DV2P04/08 viral suspension at 2.4 x 10^6^ ffu/ml. Following injection, the mice were observed daily; separate subgroups of mice were sacrificed at day 6 or 14 p.i. During mouse necropsies at day 6 and 14, specimens of serum, spleen, kidney, lung, liver, thymus, brain, and BM were collected aseptically and stored at −80 °C pending further processing. At the same time, peritoneal exudate cells (PECs) were collected by injecting 5 ml of sterile PBS-EDTA into the mouse peritoneal cavity at necropsy. Additionally, blood for serum was collected aseptically from the tails of mice on days 2, 5, and 9 p.i. to monitor viremia.

### Processing of mouse tissue and serum

Viral RNA was isolated from sera (70 μl/specimen) using the QIAmp Viral RNA Mini kit (Qiagen) and from tissue homogenates using TRIzol reagent (Life Technologies) according to the manufacturer’s protocol. Spleen, liver, kidney, thymus, lung, brain, PECs, and BM were homogenized using a Beads Crusher μT-12 (Taitec). Total RNA was extracted using TRIzol and adjusted to 200 μg/ml for use in real-time reverse transcription PCR. RNA was quantified using the One-Step SYBR PrimeScript RT-PCR kit II (Takara) and the following dengue group-specific primers: DN-F, 5’-CAATTGCTGAAACGCGAGAGAAA-3’ and–DN-R, 5’-CCCCATCTATTCAGAATCCCTGCT-3’ [26]. Each 12.5-μL reaction mixture included final concentrations of total RNA at 8 μg/mL and of each primer at 0.08 μM. For reverse transcription, the conditions were 42 °C for 5 min and 95 °C for 10 min, followed by 45 cycles of 95 °C for 5 s, 55 °C for 30 s, and 72 °C for 30 s. The results were quantified by interpopulation analysis via a standard curve generated from 10-fold serial dilutions of in vitro-transcribed DV2ChimV RNA generated using the MEGAscript Kit (Ambion). Data were analyzed with CFX Manager ver. 1.6 (Bio-Rad). To quantify vRNA derived from organs, the amounts were normalized to the total RNA from corresponding organs of mock-infected mice.

### cDNA synthesis and PCR amplification

The viral RNA isolated from the original viral stock and from the virus derived from brain or thymus virus was converted to cDNA using a One-Step SYBR Green RT-PCR Kit II (Takara) following the manufacturer’s instructions. Based on the concentrations of RNA obtained from each of the several organs, RNA samples from the brains of infected mice No. 3 and 6 and from the thymus of infected mouse No. 6 were subsequently chosen for cDNA synthesis. The details of the PCR reaction and the sequence of the primers used were as described previously [25]. The PCR products were purified by QIAquick PCR purification Kit (QIAGEN) following the manufacturer’s instruction for sequencing.

### Viral genome sequencing

Sequencing reactions were carried out by using different primers spanning the genome [25]. Following the sequencing reactions, the products were purified before loading in the plate for sequencing. The resultant sequencing products were analyzed using an ABI Prism 3130*x1* Genetic analyzer.

### Multiple sequence alignment

Multiple sequence alignment (MSA) of whole genomes among DENV reference strain 16681 (accession no.NC_001474), DV2P04/08, and viruses from brains and thymus of infected mice was carried out using GENETYX version18.0.1.

### Data deposition

The sequences described here have been deposited in the GenBank database under accession numbers LC129169 to LC129171.

## Results

### Virulence of DV2P04/08 in IFN-α/β/γR KO mice

**Fig. 1:**
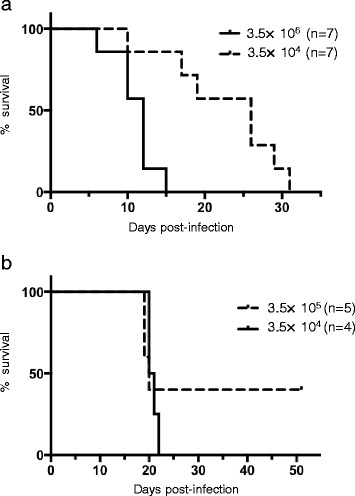
Survival analysis in IFN-α/β/γR KO mice and IFN-α/β/γR/FcγRIIB KO mice. **a** Groups of IFN-α/β/γR KO mice were intraperitoneally infected with DV2P04/08 at 3.5 × 10^6^ or 3.5 × 10^4^ ffu/mouse. **b** Groups of the IFN-α/β/γR/FcγRIIB KO mice were intraperitoneally infected with DV2P04/08 at 3.5 × 10^5^ or 3.5 × 10^4^ ffu/mouse

Nearly half of DENVs cause lethal infection to IFN-α/β/γR KO mice. These virulent DENVs are largely classified into two groups, one consisting of viruses that lead to acute lethal infection and another consisting of viruses that cause late death at about day 20 post-infection (p.i.) [24]. Most virulent DENVs belong to the latter group. To examine the virulence of DV2P04/08, IFN-α/β/γR KO mice were infected intraperitoneally with different titers of DV2P04/08 (3.5 x 10^6^ ffu and 3.5 x 10^4^ ffu). Mice infected at the higher viral titer started dying at day 6 p.i., and all of the mice in this group died before day 16 p.i. (Fig. 1a). Notably, the mice infected at the higher viral titer demonstrated a slight to marked degree of paralysis of the hind limbs at the late stage of disease. IFN-α/β/γR KO mice inoculated with a lower titer of virus manifested a slower disease course and started dying at day 10 p i., with all of the mice in this group dying by day 32 p.i. (Fig. 1a). As above, mice exhibited paralysis at the late stage of the disease (here, after day 10 p.i.). These results indicated that DV2P04/08 caused lethal infection in IFN-α/β/γR KO mice with 100% mortality rate, although mortality did not reflect acute lethal infection.

### Virulence of DV2P04/08 in IFN-α/β/γR/FcγRIIB KO mice

Antibodies (Abs) against DENV are thought to play an important role in DENV infection because the interaction between Abs and DENV can result in either immunity or enhanced virus infection by increased uptake of Ab-virus complexes thorough Fcγ receptors (FcγR) [27]. Among Fcγ receptors [28], Fcγ receptor IIB (FcγRIIB) is the only inhibitory Fcγ receptor; mice lacking FcγRIIB often exhibit autoimmune diseases [29–33]. Cross-linking of FcγRIIB by aggregation of viruses-Ab complexes may inhibit FcγR-mediated phagocytosis, which would block antibody-dependent enhancement (ADE) [34]. In our above experiment testing DV2P04/08 at two titers, survival following DENV infection is sufficiently long that mice presumably generate Abs against DENV (Fig. 1). To determine whether additional gene knockout of FcγRIIB affects symptoms in mice following DV2P04/08 infection, IFN-α/β/γR/FcγRIIB KO mice were infected intraperitoneally with different titers of DV2P04/08. The IFN-α/β/γR/FcγRIIB KO mice demonstrated slightly different patterns of survival from those seen in IFN-α/β/γR KO mice (Fig. 1b). Notably, the IFN-α/β/γR/FcγRIIB KO mice infected with 3.5 x 10^4^ ffu of virus started dying at day 20 p.i., with all of these animals dying by day 22 p.i. (Fig. 1b). Thus, infection with 3.5 x 10^4^ ffu induced 100% mortality. Mean time to death of IFN-α/β/γR/FcγRIIB KO mice was 20.5 days, which was similar to that observed for IFN-α/β/γR KO mice infected with 3.5 x 10^4^ ffu (20 days), although the IFN-α/β/γR/FcγRIIB KO mice died within shorter interval (days 20–22 p.i.) than did the IFN-α/β/γR KO mice (days 10–32 p.i.). Interestingly, 60% of IFN-α/β/γR/FcγRIIB KO mice infected with 3.5 x 10^5^ ffu died on days 19 or 20 p.i. (Fig. 1b). Thus, the mean time to death in IFN-α/β/γR/FcγRIIB KO mice was similar at the two titers, but infection at the higher titer did not yield 100% mortality. Although there was a slight difference in the survival pattern between the IFN-α/β/γR KO and IFN-α/β/γR/FcγRIIB KO mice, the difference did not reach statistical significance and the mortalities in IFN-α/β/γR/FcγRIIB KO mice also occurred within short, defined intervals (even when these KO mice were infected with different doses of virus). This observation facilitated prediction of the course of disease progression. Hence, we used IFN-α/β/γR/FcγRIIB KO animals for analysis in the subsequent experiments.

### Distribution of DV2P04/08 in DENV-infected IFN-α/β/γR/FcγRIIB KO mice

**Fig. 2:**
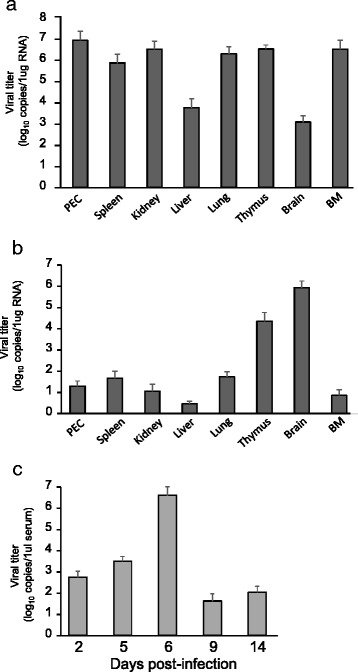
Virus titers in IFN-α/β/γR/FcγRIIB KO mice. Groups of IFN-α/β/γR/FcγRIIB KO mice were intraperitoneally infected with 2.4 × 10^6^ ffu of DV2P04/08. Organ/tissue specimens (including PEC, spleen, kidney, liver, lung, thymus, brain, and bone marrow (BM), and PECs) were collected at day 6 (**a**) or 14 (**b**) p.i. (**c**) Virus titer in mice serum at days 2, 5, 6, 9, or 14 p.i

The symptoms of DV2 P04/08-infected mice, such as the paralysis of hind limbs, appeared to be neurologic. To understand the pathogenesis of this infection, tissue distribution of the virus was examined. Since the peak of viral production previously was shown to occur at around 5–6 days p.i. in an acute systemic infection model [24], we determined the levels of vRNA in each organ at day 6 p.i. for IFN-α/β/γR/FcγRIIB KO mice infected intraperitoneally with DV2P04/08. High levels of vRNA (>10^6^ copies/μg of total RNA) were detected in peritoneal exudate cells (PECs), spleen, kidney, lung, thymus, and bone marrow (BM). Lower levels of vRNA (5.6 × 10^3^ copies/μg of total RNA) were detected in liver. Among the organs/tissues examined, the lowest levels of vRNA (1.2 × 10^3^ copies/μg of total RNA) were detected in brain (Fig. 2a). These results were similar to those obtained in mice infected with DV3P12/08 [24], which causes acute lethal systemic infection. Additional samples were obtained at day 14 p.i. from separate IFN-α/β/γR/FcγRIIB KO mice infected intraperitoneally with DV2P04/08. Notably, at day 14 p.i., brain yielded a higher titer of virus than thymus, kidney, spleen, lung, PECs, BM, or liver (Fig. 2b). Likewise, the real-time PCR results of serum virus levels at days 2, 5, 6, and 14 p.i. indicate that the peak virus titer occurred at day 6 p.i., with serum virus levels subsequently decreasing by day 14 p.i. (Fig. 2c). These results suggested that DV2P04/08 initially grew primarily in the non-neuronal organs/tissues of infected IFN-α/β/γR/FcγRIIB KO mice, with subsequent penetration into the central nervous system.

### Sequence analysis of DV2P04/08 in organs/tissues of DENV-infected IFN-α/β/γR/FcγRIIB KO mice

Since the virus levels in mouse brain were initially low but subsequently increased (in contrast to the pattern seen in non-neuronal organs and in serum), we assumed that adaptation of DV2P04/08 by mutation to a particular genomic sequence was needed to grow in the brain. To characterize this proposed sequence change, we determined the full-length genome sequences of viruses obtained at day 14 p.i. from the brains and thymuses of infected IFN-α/β/γR/FcγRIIB KO mice, and compared those sequences to those of the original (infecting) DV2P04/08 strain. While the DV2P04/08 viruses recovered from the brains and thymuses of infected mice harbored multiple mutations compared to the sequence of the parent virus, the majority of these changes represented synonymous (silent) changes in the structural and non-structural proteins encoded by the virus. However, certain mutations in the segments encoding E, NS1, NS3, NS4, and NS5 proteins represented nonsynonymous changes. Table 1 shows the amino acid (AA) residues that were polymorphic when comparing among the original DV2P04/08, viruses recovered from brain, and viruses recovered from thymus. Two mice, designated B3 and B6, that were sacrificed at day 14 p.i. were used for sequence analysis. Full-length viral genomes were successfully amplified from three specimens, specifically B3 brain, B6 thymus, and B6 brain. The original DV2P04/08, which was used to infect the IFN-α/β/γR/FcγRIIB KO mice, possessed a minor virus population encoding an E at AA 774 of the polyprotein. However, this minor population was no longer detected in any of the viruses derived from the specimens obtained at 14 days p.i. The encoded protein sequence of the major virus population recovered from B3 brain was nearly identical to that of inoculated virus itself, whereas that from B6 brain encoded 6 AA substitutions compared to the parent: T456I and S643N in E; A813S, T847A, and K949R in NS1; and T1595N in NS3. Although virus from B6 brain possessed these mutations, these changes seemed not to be necessary for viral replication in mouse brain, given that viruses from B3 brain and B6 brain did not share any of the same mutations. Interestingly, in B6 thymus, DNA sequence heterogeneity (mixture of different DNA sequences) was observed at 108 nucleotide positions (compared to nucleotide heterogeneity at 8 and 28 positions in viruses recovered from B3 brain and B6 brain, respectively) (data not shown). In B6 thymus, 11 mixtures of AA were identified, while only 3 mixtures of AA were found in B6 brain (Table 1). These results suggested greater heterogeneity of viruses recovered from the B6 thymus compared to those derived from B3 brain and B6 brain.

**Table 1:** Amino acid comparison among input DV2P04/08 and progeny viruses from mouse

Proteins	Amino acid position^a^	P04/08	B3 Brain	B6 brain	B6 thymus	16681
E	456	T	T	I^b^	T/I^b^	T
	643	S	S	N^b^	N^b^	S
	774	Q/E^b^	Q	Q	Q	Q
NS1	813	A	A	S^b^	S^b^	S^b^
	847	T	T	A^b^	T/A^b^	T
	932	D	D	D	D/N^b^	D
	949	K	K	R^b^	K/R^b^	K
NS3	1595	T	T	N^b^	T/N^b^	A^b^
	1723	R	R/K^b^	R	R/K^b^	R
	1730	E	E	E	E/K^b^	E
NS5	2494	G	G/D^b^	G	G/D^b^	G
	3125	H	H	H/R^b^	H/R^b^	H
	3135	Q	Q	Q/H^b^	Q/H^b^	Q
	3356	V	V	V/A^b^	V/A^b^	A^b^

^a^Amino acid position in polyprotein of DENV-2 16681 strain

^b^Minor amino acid variations different from those of the parental DV2P04/08 polyprotein

## Discussion

In the present study, we showed that DENV-2 clinical isolate P04/08 caused lethal infection in IFN-α/β/γR KO mice. Additional KO of the gene encoding inhibitory FcγRIIB appeared not to affect mouse survival. Deficiency of FcγRIIB is expected to increase Ab production by blocking inhibitory effects on plasma cells, amplifying the humoral immune response (as seen for increased autoimmunity in FcγRIIB KO mice) [35]. However, the effect of FcγRIIB-deficiency might have been minimized in the present study, given that the IFN-α/β/γR/FcγRIIB KO mice also lacked the IFN-γ receptor. Although our present study clearly demonstrated that DENV-2 clinical isolate P04/08 caused lethal infection in IFN-α/β/γR KO and IFN-α/β/γR/FcγRIIB KO mice, we did not detect any vascular leakage in DV2P04/08-infected mice (data not shown). This result contrasts to our recent demonstration of vascular leakage in IFN-α/β/γR KO mice infected with DV3P12/08 clinical isolate [24]. The basis for this distinction between DENV-2 and DENV-3 is currently unclear, but differences in the combination of mice and DENV serotypes or strain differences may affect such clinical manifestations.

In IFN-α/β/γR/FcγRIIB KO mice, highest production of DV2P04/08 was detected in brain at late stage of disease progression (Fig. 2). To establish infection in brain, at least two events must occur [36]. The first event is invasion into the brain; that is, the virus has to cross the blood–brain-barrier (BBB). The second event is efficient replication in neuronal cells. These two events are necessary for most pathogens, with the notable exception of the rabies virus, which is able to move into the brain via retrograde transport [37]. However, to date, the mechanism of flavivirus invasion into the brain remains unknown. We expect that the model employed here (using DV2P04/08 in receptor-KO mice) will facilitate understanding of flaviviral invasion into the brain. A key issue is the delay of virus replication in the brain (relative to that in non-neuronal organs/tissues) (Fig. 2). Specifically, DV2P04/08 was readily detected in non-neuronal organs by day 6 p.i., when the virus was present at lower levels in brain (Fig. 2a). Subsequently, viral levels rose strongly in brain to day 14 p.i., with viral replication presumably continuing to rise until the occurrence of death at around day 20 p.i. At this period, a certain degree of viral sequence diversity was observed in viruses recovered from the thymus, while greater sequence homogeneity was obtained for viruses recovered from the brain (Table 1). Although this observation reflects a small sample size, DV2P04/08 initially replicated in non-neuronal organs and eventually invaded into the central nervous system. Of note, an adaptation of DV2P04/08 was not necessary to invade brain because the sequence of viruses derived from the brain of one mouse (B3) was nearly identical to that of the infecting (parent) virus. Two scenarios are conceivable to explain the delay of virus replication in brain. In Scenario 1, the virus is unable to efficiently cross the BBB at the beginning of the infection. Thereafter, BBB permeability somehow increases at some point of disease progression, permitting invasion of the brain by the virus and subsequent viral replication. In Scenario 2, the brain is invaded at the beginning of the infection, but virus replication is impaired or permitted only in limited regions of the brain. At later time points, the virus is able to replicate explosively, presumably as the condition of mice worsens. In the mouse designated B3 in this work, both scenarios are possible because the virus recovered from the brain at day 14 p.i. was almost identical to the original infecting virus (Table 1). This virus may have succeeded in invading B3’s brain at a late stage (Scenario 1) or may have invaded at an early stage but remained suppressed in neuronal tissue pending later events (day 14 p.i.) (Scenario 2). In contrast, in the mouse designated B6 in the present work, the virus identified in brain at day 14 p.i. had many substitutions (Table 1). The substitutions were unlikely to be necessary for virus adaptation to brain because virus from mouse B3 did not harbor these mutations. This mutant virus is likely to be produced in non-neuronal organs because this mutant virus existed in thymus (Table 1). In thymus, we found a diverse population of viral sequences, including that of the original virus. By day 14 p.i., the infected mice should have been producing neutralizing Abs. The observed diversity of virus in thymus is presumably due to escape from these immunological pressures, although we failed to detect any differences in antibody titiers between B3 and B6 mice (Additional file 1). In the mouse B6, virus seemed to invade neuronal tissue at late stage (Scenario 1).

Unfortunately, we were unable to amplify viral sequences from other (non-thymus, non-brain) organs of the infected animals, precluding sequence analysis of the virus replicating in other organs and tissues. Nevertheless, our result suggests the following hypothesis to explain emergence of mutant viruses in the brain of B6. During the first 5–6 days p.i., DV2 P04/8 replicated in non-neuronal organs. Subsequently (during days 7–13 p.i.), the mouse immune system reacted to the infecting virus and nearly succeeded in clearing virus from non-neuronal organs, with the exception of the thymus. During this interval, the virus started to escape in non-neuronal organs, as evidenced by the diverse viral population in the thymus. Some of the viral population succeeded in escaping from immune pressure, permitting invasion of the brain and maximum viral replication, and resulting in death. We postulate that there must be a release of some unidentified factor from the infected organs to increase the permeability of the BBB at some point. Presumably, only a selected subpopulation of escaping mutant virus produced in other organs succeeds in reaching the brain. However, the true scenario might not be that simple because there was no sequence variation among virus derived from B3 mouse. We cannot exclude other possibilities to explain the sequence variation of virus observed in B6 mouse.

Further study will be needed to elucidate the precise molecular mechanisms for DENV neurovirulence. Notably, we will need to clarify the roles of pathogenesis, neurovirulence, and vascular leakage on viral replication, invasion of specific organs/tissues, and evasion of immune regulation. These distinctions will be critical to better understanding of severe DENV manifestations, including DHF and DSS.

## Conclusions

In summary, DV2P04/08 easily invade into mouse brain and caused lethal infection. IFN-α/β/γR KO mice nearly succeeded in eliminating virus from non-neuronal organs but could not avoid brain invasion. Mutant virus detected in B6 mouse brain was probably the result of escape from mouse immune system and produced in non-neuronal organs such as thymus. Our study provides new insights into the dynamics of viral evolution in individuals.

## Additional file

Additional file 1: Vero cells were infected with DV2P04/08 and cultured for 24 h. The cells were fixed with 3.3% formaldehyde and permeabilized with 1% Triton X-100. The cells were reacted with diluted serum (1:160 dilution) of DV2P04/08–infected mice. Anti-dengue virus monoclonal antibody 4G2 or uninfected mouse serum was used as a positive or a negative control, respectively. (PPTX 8907 kb)

## Abbreviations

AA: Amino acid
Abs: Antibodies
ADE: Antibody dependent enhancement
B3: Mouse no. 3
B6: Mouse no.6
BBB: Blood brain barrier
BM: Bone marrow
C: Capsid
DENV: Dengue virus
DF: Dengue fever
DHF: Dengue haemorrhagic fever
DSS: Dengue Shock Syndrome
DV2: Type 2 dengue virus
E: Envelope
ffu: Focus forming units
IFN: Interferon
KO: Knock out
M: Membrane
MSA: Multiple sequence alignment
NS: Nonstructural
p.i.: Post infection
PEC: Peritoneal exudate cells
UTR: Untranslated Regions
α: Alpha
β: Beta
γ: Gamma
