# Supplementary material for: Sequence diversity of dengue virus type 2 in brain and thymus of infected interferon receptor ko mice: implications for dengue virulence
Source: Virol J. 2016 Nov 30;13:199. doi: 10.1186/s12985-016-0658-4 (PMC5129197; doi:10.1186/s12985-016-0658-4)
Supplement: Additional file 1: — Vero cells were infected with DV2P04/08 and cultured for 24 h. The cells were fixed with 3.3% formaldehyde and permeabilized with 1% Triton X-100. The cells were reacted with diluted serum (1:160 dilution) of DV2P04/08–infected mice. Anti-dengue virus monoclonal antibody 4G2 or uninfected mouse serum was used as a positive or a negative control, respectively. (PPTX 8907 kb) [file 12985_2016_658_MOESM1_ESM.pptx]

## Slide 1
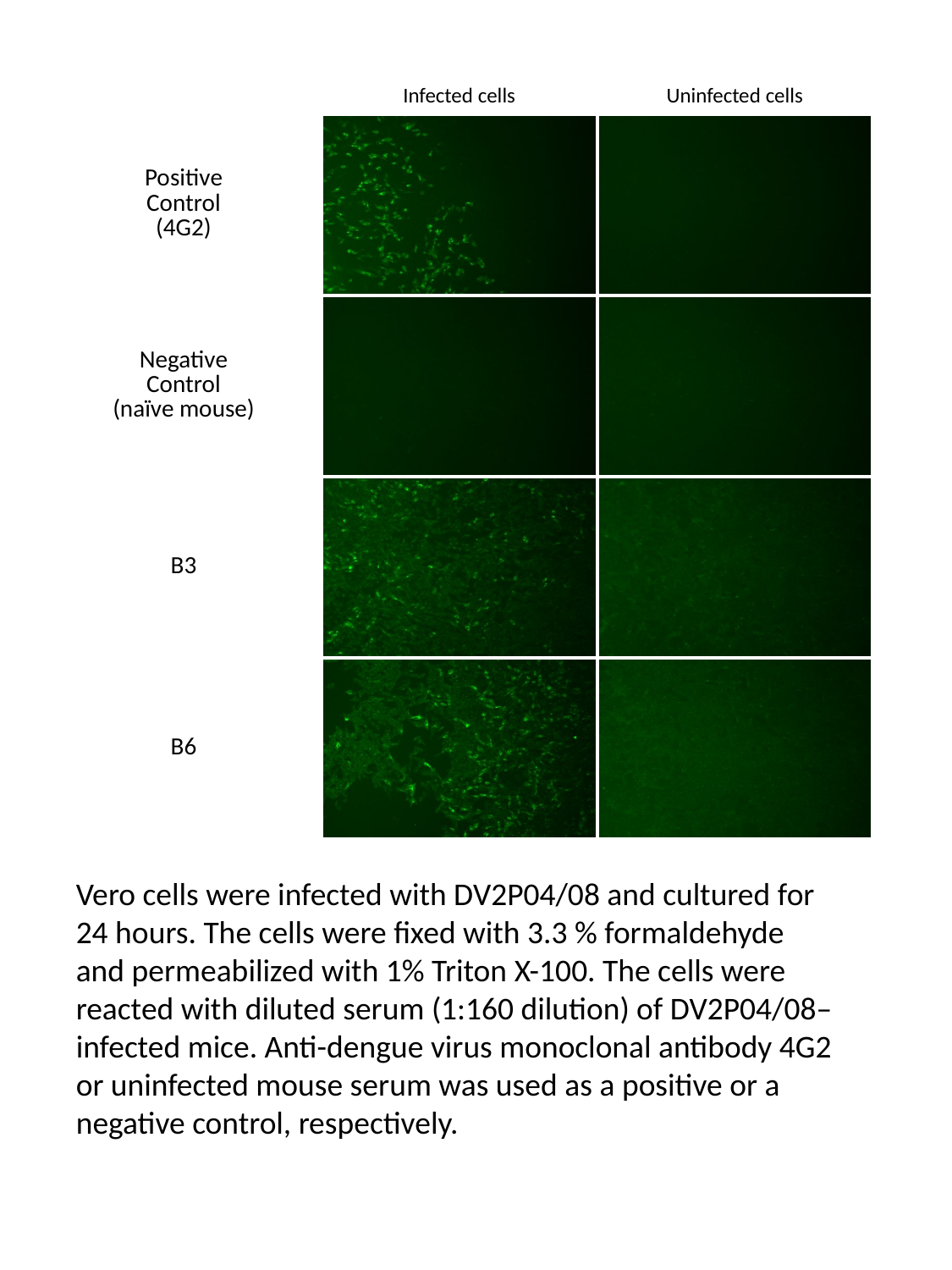

| | Infected cells | Uninfected cells |
| --- | --- | --- |
| Positive Control (4G2) | | |
| Negative Control (naïve mouse) | | |
| B3 | | |
| B6 | | |
Vero cells were infected with DV2P04/08 and cultured for 24 hours. The cells were fixed with 3.3 % formaldehyde and permeabilized with 1% Triton X-100. The cells were reacted with diluted serum (1:160 dilution) of DV2P04/08–infected mice. Anti-dengue virus monoclonal antibody 4G2 or uninfected mouse serum was used as a positive or a negative control, respectively.
